# A Structured Approach to Involve Stakeholders in Prioritising Topics for Systematic Reviews in Public Health

**DOI:** 10.3389/ijph.2024.1606642

**Published:** 2024-08-21

**Authors:** Dyon Hoekstra, Margot Mütsch, Annegret Borchard, Christina Kien, Ursula Griebler, Erik Von Elm, Eva Rehfuess, Ansgar Gerhardus, Stefan K. Lhachimi

**Affiliations:** ^1^ Research Group for Evidence-Based Public Health, Leibniz-Institute for Prevention Research and Epidemiology (BIPS), Institute for Public Health and Nursing Research (IPP), University of Bremen, Bremen, Germany; ^2^ Health Sciences Bremen, University of Bremen, Bremen, Germany; ^3^ Department of Special Needs Education and Rehabilitation, University of Oldenburg, Oldenburg, Germany; ^4^ Department of Epidemiology, Institute of Epidemiology, Biostatistics and Prevention, Faculty of Medicine, University of Zurich, Zurich, Switzerland; ^5^ Cochrane Switzerland Center for Primary Care and Public Health, University Center of General Medicine and Public Health, Lausanne, Switzerland; ^6^ Department for Evidence-Based Medicine and Evaluation, University for Continuing Education Krems (Danube University Krems), Krems an der Donau, Austria; ^7^ Cochrane Austria University for Continuing Education Krems, Krems an der Donau, Austria; ^8^ Institute for Medical Information Processing, Biometry, and Epidemiology, Faculty of Medicine, Ludwig Maximilian University of Munich, Munich, Germany; ^9^ Pettenkofer School of Public Health, Munich, Germany; ^10^ Department for Health Services Research, Institute for Public Health and Nursing Research, University of Bremen, Bremen, Germany; ^11^ Department of Nursing Management, University of Applied Sciences Neubrandenburg, Neubrandenburg, Germany

**Keywords:** public health, systematic review, priority setting, Delphi technique, stakeholder involvement

## Abstract

**Objectives:**

This study aimed to develop and apply a structured approach for prioritising topics for systematic reviews in public health, framed according to the readily applicable PICO format, which encourages the involvement of stakeholders’ preferences in a transparent matter.

**Methods:**

We developed a multi-stage process, consisting of a scoping and two Delphi stages with web-based surveys and invited public health stakeholders in Switzerland to participate: First, respondents specified topics for different public health domains, which were reformulated in a PICO format by content analysis. Second, respondents rated the topics using five stakeholder-refined assessment criteria. Overall rankings were calculated to assess differences between stakeholder groups and rating criteria.

**Results:**

In total, 215 respondents suggested 728 topics altogether. The response rate in the two Delphi stages was 91.6% and 77.6%, respectively. Most top-rated review topics focused on the effectiveness of interventions providing education to different target groups, followed by interventions to increase access to specific healthcare services.

**Conclusion:**

Our approach encourages involvement of stakeholders in identifying priorities for systematic reviews and highlights disparities between stakeholders and between individual criteria.

## Introduction

A major challenge in health research is that demand for funding usually surpasses the available resources. Health organisations carrying out or funding research must choose which research is to be prioritised [1]. Health research are often selected based on the priorities of researchers or a select group of experts, leaving out the interests of other relevant stakeholders, such as patients, policymakers and healthcare professionals [2–4]. Consequently, funded research can be of low priority to these potential users of research, making investments less efficient and increasing the risk of research waste [2, 5, 6]. Likewise, research needs of relevant stakeholders may not be addressed by funded research if they are not prioritised [7].

Several studies indicated that researchers sometimes overlook research needs of stakeholders in research priority setting (RPS) [4–6]. For example, patients or healthcare professionals prioritised non-drug treatments or management of social and emotional issues higher as researchers [8–10]. Recently, the interests of different stakeholders in health have been considered with varying degrees of involvement when determining what health research should be considered a priority [4]. This has led to a growing demand for structured processes for prioritising health research in which distinct stakeholder groups are involved [5, 11–13].

Furthermore, opinions, experiences and interests of individual stakeholders or the stakeholder groups they represent, lead to divergent views on which research areas should be considered worthy of priority [14]. This can lead to potential conflicts in decision-making [2, 9, 15]. Hence, a wide range of stakeholders and interest groups can utilise results from a structured RPS study as a valid basis for discussion [12, 13, 16]. Using a transparent process, can also make it easier for stakeholders to accept decisions that do not align with their own interest [17].

Involving a broad range of stakeholders in an RPS study that specifically focuses on public health is particularly challenging due to the multifaceted nature of the field, crossing different sectors and different levels, each containing a variety of sometimes hard to define and reach target groups. A few RPS studies have aimed to prioritise topics solely in the field of public health (e.g., [18–22]), with varying degrees of structuring and stakeholder involvement.

Additionally, the multifaceted character of public health poses a challenge when prioritising for evidence synthesis by conducting systematic reviews. Systematic reviews synthesize evidence from various studies, producing a type of evidence that stand out due to their methodological rigor, comprehensive coverage, structured synthesis, and significant role in evidence-based decision making, which makes them a highly valuable and reliable source of evidence in research, healthcare, policy-making, and other fields [23]. Systematic reviews are important for generating evidence-based answers to research questions, often about the effects of interventions or measures regarding a specific target group, by reviewing available scientific literature. Systematic reviews can offer a holistic view that can address the complexity that is often involved in public health issues and can therefore provide an evidence base for developing and implementing public health policies and programs [24].

Cochrane is an international network that prepares, maintains, and promotes the accessibility of systematic reviews. Although groups within Cochrane have used various methods for prioritising topics for systematic reviews in their research area, an inclusive, transparent and structured process to prioritise review topics is still lacking [19, 25, 26].

Furthermore, involving various stakeholders causes a challenge when prioritising systematic review topics because of the initial difficulty to develop specific systematic review questions, for example, based on the PICO framework. PICO stands for Population, Intervention, Comparator and Outcome [27] and is often used as a framework to formulate a systematic review question and to define inclusion and exclusion criteria for the review [27]. Since not all stakeholders know how to process or develop PICO-formatted questions or topics, an RPS for prioritising systematic review topics needs to enable this [28].

Therefore, we–on behalf of Cochrane Public Health Europe (CPHE), a subdivision of Cochrane Public Health–aimed to develop and apply a structured RPS approach in Switzerland that enables all stakeholders to prioritise systematic review topics for public health research.

We sought to investigate potential similarities and differences among the priorities of different stakeholder groups and aimed to examine which assessment criteria are considered most important for prioritising systematic review topics according to different stakeholder groups.

The study was approved by the ethical review board of the University of Bremen in Germany and the Canton of Zurich in Switzerland and we reported it here according to the REPRISE guideline [5].

## Methods

For this study we used a multi-stage process, comprising a scoping stage and two Delphi stages:

In the scoping stage, we first established an advisory board of three subject-matter and methodological experts from Switzerland, Austria, and Germany. The advisory board members reviewed the study design and provided valuable insights for setting the scope of the study [29]. As such, three relevant domains of public health research based on the classification of the European SPHERE project [20] were identified: prevention, health promotion, and health services.

Based on these public health domains the following stakeholder groups have been deemed relevant:

• Research and/or higher education
• Administration and/or politics
• Health organisations representing certain target groups in the population
• Organisations representing healthcare professionals and institutions
• Health insurers

Conducting desk-based searches and using recommendations from the research team and the advisory board, we identified organisations in Switzerland within each stakeholder group. We then invited each organisation (149 in total) to nominate respondents from within their organisations to participate in the Delphi rounds. Of the 149 stakeholder organisations invited, 70 (47%) organisations nominated a total of 215 individuals (see Table 2).

Next, we applied a modified Delphi consisting of two online Delphi rounds for the prioritisation of the review topics. We used Lime Survey [30] for the online surveys (available in German, French and Italian).

### Delphi Round 1

In the first Delphi round (March/April 2018), respondents were asked to propose up to five topics for systematic reviews which they thought to be relevant. To accommodate the varying familiarity of the stakeholders with systematic reviews, we asked them to specify their proposed review topics according to the different components of the PICO format in a stepwise manner by providing a template for each component. We did not ask the respondents to suggest a comparator (C), as this was deemed too challenging for respondents, most of whom had never worked with systematic reviews.

We asked the respondents to propose review topics across the three pre-specified domains of public health, i.e., prevention, health promotion, and health services. Two people (DH and MM) coded and aggregated the proposed topics using content analysis in the software MAXQDA [31] while a third person (SL) provided feedback. The aggregation through the content analysis ensured that the large number of redundant or overlapping suggestions were transferred in a practicable amount of sufficiently distinct review topics. We counted how many review topics were proposed in each public health domain to understand which target groups (population), interventions, and outcomes were considered most frequently.

Additionally, respondents had to select criteria that they considered most important for assessing topics for systematic reviews. For establishing an initial list of criteria, criteria taken from literature were discussed within the research team and with the advisory board. Based on the initial list, respondents could propose further assessment criteria, which we aggregated, and if applicable merged, with the initial list of criteria. We then examined which assessment criteria were selected most frequently. But we also ensured that the criteria used for rating, measured distinct dimensions to be able to sufficiently discriminate in the assessment of the review topics. Table 1 shows the five stakeholder-refined assessment criteria that were considered most useful for rating review topics in public health.

**TABLE 1:** List of stakeholder-refined assessment criteria after Delphi round 1 (Switzerland, 2024).

	Assessment criteria	Operational definition
A	Improving the health of the population	Systematic reviews regarding the topic can lead to measures that contribute to improving the health of the population (e.g., reducing the burden of disease, promoting physical and mental health)
B	Health equity	Systematic reviews regarding the topic can lead to measures that contribute to more health equity
C	Insufficient research to date	Systematic reviews regarding the topic may indicate that research results on this topic are insufficient (e.g., are not available, are of insufficient quality, are not up to date or will become more important in the future)
D	Effect on public health if successful	Systematic reviews regarding the topic can have a positive effect on further research or practice in public health
E	Potential for innovative action	Systematic reviews regarding the topic have a high potential for innovative findings

Finally, we asked respondents to rate their previous experience with and their attitude towards systematic reviews.

### Delphi Round 2

All those nominated for participation in the first Delphi round were invited to participate in the second Delphi round (August/October 2020), independent of whether they had responded to the first round. Respondents were asked to rate the review topics based on the content analysis by applying the stakeholder-refined assessment criteria on a scale from 1 “totally agree” to 4 “totally disagree.” Each respondent had to assess approximately only one-third of the review topics, which were randomly assigned.

In this round, we additionally asked respondents to weigh the importance of the five stakeholder-refined assessment criteria on a 100-point scale.

We calculated the mean for each topic (named the “rating score”) for all five assessment criteria combined and for the five assessment criteria separately, to rank the topics accordingly [32]. Furthermore, the inclusion of a variety of stakeholder groups allowed us to compare the rating and ranking of the review topics and the criteria weighting by stakeholder group.

We used IBM SPSS Statistics (Version 23) software for data management and analysis.

## Results

### Characteristics of Participating Stakeholders

Of the 215 nominees, 197 (91.6%) started the online survey for the first Delphi round and 133 (67.5%) completed it (see Table 2). On average, the respondents took 25.5 min to complete the survey for the first Delphi round. Of the 197 respondents, 160 (81%) responded in German, 29 (15%) in French and 8 (4%) in Italian.

**TABLE 2:** Participation of organisations in nomination and of individuals in the Delphi rounds, by stakeholder group (Switzerland, 2024).

Stakeholder group	Invitation to nominate respondents for Delphi rounds I & II	Nomination of respondents for Delphi rounds I & II	Delphi round I	Delphi round II
	Nr. of organisations	Nr. of organisations	Nr. of respondents	Nr. of respondents
Research and/or higher education	28 (18.8%)	26 (37.1%)	77 (39.1%)	69 (41.3%)
Representatives of general public/patients	49 (32.9%)	15 (21.4%)	36 (18.3%)	39 (23.4%)
Administration and/or politics	35 (23.5%)	16 (22.9%)	37 (18.8%)	33 (19.8%)
Representatives of healthcare professionals/institutions	25 (16.8%)	9 (12.9%)	17 (8.6%)	15 (9.0%)
Health insurers	12 (8.1%)	4 (5.7%)	1 (0.5%)	3 (1.8%)
Other	—	—	11 (5.6%)	7 (4.2%)
Missing	—	—	*18 (9.1%)*	*1 (0.6%)*
Total	**149 (100%)**	**70 (100%)**	**197 (100%)**	**167 (100%)**

The values in the last row show the total number of organisations that were invited, the total number of organisations that participated, the total number of respondents that participated in Delphi round 1, and the total number of participants that participated in Delphi round 2.

A majority of respondents in the first Delphi round described their work as being primarily “research and/or teaching” (n = 77, 39.1%). Followed by “administration and/or politics” (n = 37, 18.8%), “health organisations representing certain target groups in the population” (n = 36, 18.3%), “representatives of healthcare professionals and institutions” (n = 17, 8.6%); “health insurers” (n = 1, 0.5%); and “other” (n = 11, 5.6%).

In total 167 (77.7%) individuals participated in the second round, of which 128 (76.6%) completed the online survey. The respondents took 19.5 min on average to complete the survey for the second Delphi round. Only four respondents (2%) mentioned that they had not participated in the first round. Most respondents indicated that their field of activity was in “research and/or teaching” (n = 69, 41.3%), then “health organisations representing certain target groups in the population” (n = 39, 23.4%); “administration and/or politics” (n = 33, 19.8%); “representatives of healthcare professionals and institutions” (n = 15, 9.0%); health insurers (n = 3, 1.8%); and “other” (n = 7, 4.2%).

### Respondents’ Experience With Systematic Reviews

Most of the respondents who answered the set of questions regarding their experience with systematic reviews (n = 138), stated some familiarity with systematic reviews and considered them essential for research: They knew what systematic reviews meant (n = 122, 88%) and had used them in their professional activities (n = 109, 79%). Two-thirds of the respondents relied on Cochrane for systematic review results (n = 91, 66%). A majority also agreed with the importance of systematic reviews in public health research (n = 84, 61%) and practice (n = 87, 63%); about half found the results of systematic reviews helpful for their own work (n = 69, 50%). Furthermore, approximately half of the respondents knew how to find relevant systematic reviews (n = 80, 58%) and less than half of the respondents (n = 59, 43%) indicated that they do *not* have difficulties understanding the essence of systematic reviews. Finally, less than half (n = 58, 42%) of the respondents had been involved in the process of producing a systematic review.

### Results of the Content Analysis

The respondents suggested a total of 728 review topics in the first Delphi round that were compiled in a comprehensive list of 245 review topics, which we further aggregated into 27 categories across the three domains (see Table 3). For example, in the domain “prevention,” most suggested topics (n = 32) were assigned to the category “Interventions for screening and/or monitoring measures,” comprising often mentioned suggestions such as “More early screening to detect developmental abnormalities.”

**TABLE 3:** Frequency table of categories after content analysis (Switzerland, 2024).

Domain: Prevention (331 suggestions → 207 excluded as “no review topic”)			Domain: Health promotion (211 suggestions → 110 excluded as “no review topic”)		Domain: Health service (186 suggestions → 104 excluded as “no review topic”)	
	Category	Number of suggestions	Category	Number of suggestions	Category	Number of suggestions
1	Interventions on screening/monitoring	32	Interventions to strengthen health literacy	27	Interventions regarding (holistic) care	14
2	Interventions to strengthen health literacy	21	Interventions for health education	16	Interventions to strengthen health literacy	14
3	Interventions for health education	16	Interventions to improve a healthy diet	14	Interventions to improve professionalism and networking in the healthcare system	10
4	Interventions regarding the participation of specific target audience	13	Interventions to improve the health system	14	Interventions to improve the health infrastructure	9
5	Interventions to improve the healthcare system	13	Interventions to improve movement behaviour	12	Interventions to improve access to healthcare services	9
6	Interventions to improve a healthy diet	10	Interventions to improve the living condition of specific target audience	10	Interventions to improve the provision in the healthcare system	8
7	Interventions to improve living conditions	7	Interventions related to medical interventions and treatments	4	Interventions to improve treatment approaches and methods	7
8	Interventions related to medical or physical interventions	6	Interventions to reduce addiction	2	Interventions to improve the communication between doctors and patients	6
9	Interventions to improve movement behaviour	4	Interventions on screening/monitoring	2	Interventions on screening/monitoring	5
	Total	124		101		82

	No intervention - total	207 (63%)	No intervention - total	110 (52%)	No intervention - total	104 (56%)
11	No intervention – suggestion is more like an open problem or question statement	95	No intervention – suggestion is more like an open problem or question statement	41	No intervention – suggestion is more like an open problem or question statement	42
12	No intervention – suggestion is too unspecific or additional PICO components are missing	92	No intervention – suggestion is too unspecific or additional PICO components are missing	51	No intervention – suggestion is too unspecific or additional PICO components are missing	46
13	No intervention – suggestion is more like an outcome	20	No intervention – suggestion is more like an outcome	18	No intervention – suggestion is more like an outcome	16

We excluded 421 suggestions for review topics because they were: 1) defined as open questions or general statements (e.g., “Minimal case numbers: Quality feature or incentive for overtreatment?”), 2) too unspecific or not presented in PICO format (e.g., Development of new task allocations), or 3) defined only as outcomes (e.g., “Reduction of infant and maternal mortality”).

Although there was some overlap between the suggestions in the different domains of public health, the majority of suggestions for review topics could clearly be allocated to a single domain.

### Rating of Review Topics

The overall rating results for all review topics and the 15 best-rated review topics per criterion, can be found in Supplementary Files 1, 2. The 15 best-rated review topics overall can be found in both Table 4, 5.

**TABLE 4:** Ranking differences between criteria for the top 15 review topics (Switzerland, 2024).

	Overall		A. Improving the health of the population		B. Health equity		C. Insufficient research to date		D. Effect on public health if successful		E. Potential for innovative action	
Review topic (total number of times assessed)	R	M	R	M	R	M	R	M	R	M	R	M
Improved access to prevention services for specific target groups (159)	**1**	**1.47**	3	1.41	2	1.21	5	1.48	48	1.88	2	1.37
Cognitive training against dementia diseases (156)	**2**	**1.63**	8	1.48	26	1.59	1	1.32	85	2.11	15	1.63
Highlight target-group-specific good practices in vulnerable groups (163)	**3**	**1.64**	37	1.73	1	1.19	87	2.06	12	1.50	20	1.72
Better education for relatives of people with a mental illness (166)	**4**	**1.65**	11	1.55	7	1.38	9	1.57	98	2.19	18	1.67
Mental health education for adults (165)	**5**	**1.66**	15	1.59	118	2.05	4	1.47	16	1.53	10	1.59
More education about e-cigarettes/oral tobacco (SNUS) for children and adolescents (156)	**6**	**1.67**	1	1.34	4	1.30	44	1.84	58	1.97	37	1.88
Peer-to-peer education on health risks from drugs (171)	**7**	**1.69**	96	1.91	58	1.77	46	1.84	17	1.56	4	1.44
Integration with equal opportunities of chronically ill people into society (157)	**8**	**1.71**	57	1.81	60	1.79	20	1.70	2	1.36	33	1.87
Information in schools about the correct use of mobile phones (167)	**9**	**1.72**	18	1.62	57	1.77	49	1.86	28	1.69	17	1.66
Improving health literacy of nursing home staff in taking care of elderly people with mental disorders (158)	**10**	**1.72**	9	1.52	86	1.88	74	2.00	19	1.57	7	1.55
Provide healthy food in schools and workplaces (170)	**11**	**1.75**	21	1.63	62	1.79	57	1.91	38	1.79	16	1.65
Improved education about sexual health for specific target groups (162)	**12**	**1.75**	6	1.45	10	1.39	21	1.70	138	2.38	39	1.90
Better access to adequate care for marginalized groups (169)	**13**	**1.76**	17	1.62	15	1.46	31	1.74	150	2.44	11	1.59
Increased early recognition structures of mental illnesses (136)	**14**	**1.78**	14	1.59	120	2.06	52	1.88	11	1.50	13	1.61
Mental health resilience training (161)	**15**	**1.79**	10	1.54	67	1.81	3	1.46	149	2.43	25	1.79
Better access to good quality clinics for vulnerable groups in poor countries (158)			113	1.97	49	1.73	201	2.56	**1**	1.32	234	3.07
Support for group practices with interprofessional cooperation (163)			5	1.44	114	2.03	43	1.84	207	2.72	**1**	1.35
Mean (of all 245 review topics)		**2.21**		**2.12**		**2.13**		**2.22**		**2.27**		**2.33**

R, rank; M, mean.

The bold values in the first two columns stipulate the overall rank and mean of the top-15 rated topics for all criteria combined. The values in the following columns present the rank and mean of these topics by criteria. The final row highlights the mean of the rating of all 245 topics.

**TABLE 5:** Ranking differences between stakeholder groups for the top 15 review topics (Switzerland, 2024).

	Overall (n = 167)		Research and/or higher education (n = 69)		Representatives of the general public/patients (n = 39)		Administration and/or politics (n = 33)		Representatives of healthcare professionals/institutions (n = 15)		Health insurers (n = 3)		Other (n = 7)	
Review topic (total number of times assessed)	R	M	R	M	R	M	R	M	R	M	R	M	R	M
Improved access to prevention services for specific target groups (159)	**1**	**1.47**	1	1.55	1	1.42	5	1.64	10	1.48	1	1.00	1	1.00
Cognitive training against dementia diseases (156)	**2**	**1.63**	3	1.60	17	1.74	8	1.68	34	1.67	—	—	1	1.00
Highlight target-group-specific good practices in vulnerable groups (163)	**3**	**1.64**	18	1.77	4	1.53	10	1.76	5	1.37	1	1.00	17	1.75
Better education for relatives of people with a mental illness (166)	**4**	**1.65**	10	1.71	11	1.64	13	1.81	33	1.67	12	2.00	34	2.20
Mental health education for adults (165)	**5**	**1.66**	20	1.77	3	1.50	6	1.64	11	1.50	12	2.00	28	2.00
More education about e-cigarettes/oral tobacco (SNUS) for children and adolescents (156)	**6**	**1.67**	12	1.73	10	1.62	12	1.80	207	2.50	16	2.50	10	1.50
Peer-to-peer education on health risks from drugs (171)	**7**	**1.69**	1	1.55	18	1.75	27	1.94	192	2.40	6	1.75	7	1.33
Integration with equal opportunities of chronically ill people into society (157)	**8**	**1.71**	6	1.63	16	1.71	9	1.70	39	1.72	10	2.00	10	1.50
Information in schools about the correct use of mobile phones (167)	**9**	**1.72**	25	1.79	24	1.78	3	1.61	18	1.56	1	1.00	7	1.33
Improving health literacy of nursing home staff in taking care of elderly people with mental disorders (158)	**10**	**1.72**	32	1.84	46	1.89	7	1.64	3	1.33	10	2.00	44	2.50
Provide healthy food in schools and workplaces (170)	**11**	**1.75**	5	1.62	35	1.83	26	1.94	129	2.10	—	—	23	1.88
Improved education about sexual health for specific target groups (162)	**12**	**1.75**	61	1.98	15	1.67	5	1.64	10	1.48	10	2.00	14	1.60
Better access to adequate care for marginalized groups (169)	**13**	**1.76**	29	1.83	20	1.76	13	1.81	74	1.88	5	1.67	56	2.75
Increased early recognition structures of mental illnesses (136)	**14**	**1.78**	30	1.83	26	1.78	74	2.11	37	1.71	5	1.67	10	1.50
Mental health resilience training (161)	**15**	**1.79**	38	1.88	36	1.84	20	1.88	42	1.73	9	1.89	35	2.21
Improving the user-friendliness of digital health information for older people aged 80 and over (170)			26	1.82	28	1.78	**1**	1.53	21	1.59	212	4.00	50	1.67
Educating doctors and pharmacists about obesity (162)			49	1.91	55	1.92	184	2.53	**1**	1.24	29	1.83	17	1.30

R, rank; M, mean.

The bold values in the first two columns stipulate the overall rank and mean of the top-15 rated topics. The values in the following columns present the rank and mean of these topics as rated by the different stakeholder groups. The final two rows highlight the rank and mean of two topics that were ranked best by certain stakeholder groups, but that are not among the overall top-15 ranked topics.

Among the top 15 review topics, most focus on interventions providing education to different target groups, followed by interventions to increase access to specific healthcare services. Mental health and substance use were the public health topics prioritised most often. Finally, the target groups considered most frequently are children and adolescents, specific target groups, and vulnerable groups.

We found large differences in the rating of review topics when examining the five assessment criteria separately. Of the 15 best rated review topics, not a single one was ranked in the top 15 in all five criteria individually. For example, the overall top priority “Improved access to prevention services for specific target groups” was ranked 3rd, 2nd, 5th, and 2nd, respectively, according to the criteria “Improving the health of the population,” “Health equity,” “Insufficient research to date,” and “Potential for innovative action”; however, it was ranked only 48th according to the criterion “Effect on public health if successful.”

Even larger differences in the ranking per criterion can be observed for many review topics from the top 15 list (see Table 4). For example, only four review topics that are in the top 15 according to the criterion “Insufficient research to date” are also represented in the overall top 15 review topics. Furthermore, the highest ranked review topics for the criteria “Effect on public health if successful” and “Potential for innovative action” are not at all represented in the overall top 15 review topics list and are even ranked among the worst 15 review topics according to two other criteria (i.e., “Insufficient research to date” and “Effect on public health if successful”).

Likewise, large differences in the rankings can be observed when comparing the overall rating between different stakeholder groups (see Table 5). Our results show that only the review topic with the best overall rating was rated in the top 15 by each stakeholder group. For all other review topics in the overall top 15 we found at least one stakeholder group that did not rate it in their top 15 list. However, the differing stakeholder group was not for every topic the same one, so none of the individual stakeholder groups was solely responsible for the observed differences.

The importance of using different assessment criteria and different stakeholder groups is further highlighted by the weighting of the criteria (see Table 6). Across all respondents, the criterion “improving the health of the population” received the highest weight of 29.49 (out of 100). The criterion “effect on public health if successful” was weighted lowest with 12.28.

**TABLE 6:** Weighting of assessment criteria by stakeholder group (Switzerland, 2024).

	Criterion									
	A. Improving the health of the population		B. Health equity		C. Insufficient research to date		D. Effect on public health if successful		E. Potential for innovative action	
Stakeholder group	M	SD	M	SD	M	SD	M	SD	M	SD
Research and/or higher education (n = 69)	29.65	1.89	22.12	1.80	18.07	1.64	12.40	1.21	17.77	1.49
Representatives of the general public/patients (n = 39)	36.43	3.82	22.86	3.64	15.71	3.30	12.50	2.43	12.50	3.00
Administration and/or politics (n = 33)	32.12	2.80	19.62	2.67	15.39	2.42	11.92	1.78	20.96	2.21
Representatives of healthcare professionals/institutions (n = 15)	25.11	2.38	25.61	2.27	19.44	2.06	12.17	1.52	17.67	1.87
Health insurers (n = 3)	15.00	10.09	25.00	9.62	15.00	8.74	15.00	6.43	30.00	7.95
Other (n = 7)	33.33	8.24	11.67	7.85	20.00	7.14	11.67	5.25	23.33	6.49
Overall (n = 167)	**29.49**	**14.53**	**22.44**	**13.60**	**17.68**	**12.24**	**12.28**	**8.94**	**18.11**	**11.37**

The respondents weighted the importance of each criterion for assessing the systematic review topics by awarding a total of 100 points across the five criteria. This table shows the mean (M) and standard deviation (SD) of these weightings by stakeholder group. The last row shows the mean and standard deviation of the weighting for all stakeholder groups combined.

The high standard deviation of the overall weights per criterion (varying from 8.94 to 14.53) is mainly due to the large variability in the weightings of the different stakeholder groups. For example, (see also Table 6): Respondents from “research and/or education” (M = 29.65, SD = 1.89), “representatives of health professionals/institutes” (M = 36.43, SD = 3.82) and from “administration and/or politics” (M = 32.12, SD = 2.80) assigned the highest weight to the criterion “improving the health of the population”; whereas “Health insurers” weighted this criterion the lowest (M = 15.00, SD = 10.09). In addition, “health organisations representing certain target groups in the population” weighted the criterion “health equity” highest (M = 25.61, SD = 2.27), whereas “health insurers” rated the criterion “potential for innovative action” the highest (M = 30.00, SD = 7.95).

## Discussion

We employed a multi-stage RPS approach, integrating a scoping stage and two Delphi stages with web-based surveys, to involve a broad range of stakeholders in Switzerland in the prioritisation process. The approach was developed on behalf of Cochrane Public Health Europe (CPHE), a subdivision of Cochrane Public Health, and was applied in Switzerland to prioritise systematic review topics for public health research. We intentionally limited the Delphi process to two rounds, contrary to many Delphi studies conducting at least three rounds [33]. Therefore, the results are an indication of preferences rather than a true consensus.

Several RPS studies and approaches focused on systematic reviews in health research in general or in certain fields within health research, with varying degrees of stakeholder involvement and use of assessment criteria [34, 35]. However, our structured RPS approach is developed to determine and prioritise systematic reviews solely for public health topics, which particularly 1) allows for the participation of a large panel of respondents from all relevant stakeholder groups in easy-to-conduct and easy-to-replicate online surveys, 2) implements assessment criteria specifically for public health and by public health stakeholders to allow for intra-criteria comparisons, and 3) assists all stakeholders in developing relevant systematic review topics in a PICO format before the prioritisation exercise and without needing prior expertise on systematic reviews.

In order to get a robust insight of what evidence needs exist in public health and not be limited only to the insights of a small group of experts or decision-makers, we aimed for a multi-perspective approach in our RPS study [4]. Therefore, after careful scoping to include all relevant stakeholder groups for this study, we applied a recruitment strategy that encouraged invited organisations to self-select respondents from within their own organisation to increase the likelihood that those invited will participate [36].

Due to our recruitment strategy and the accessibility of our RPS study as an anonymised online survey design we managed not only to recruit different stakeholders, we also achieved high participation rates in the first and second Delphi round of 91.6% and 77.7%, respectively (Cf. [37–39]). This represents a very high participation rate and demonstrates that with our approach it is feasible to involve many stakeholders in a structured RPS study using limited resources.

Applying our approach, we identified five stakeholder-refined assessment criteria that were deemed most important by the respondents for assessing topics for systematic reviews; albeit with differences in how these are weighted by different stakeholder groups. Previous RPS studies have listed assessment criteria that can be used in RPS and highlighted the importance of selecting multiple assessment criteria that fit to the specific context and that can sufficiently discriminate between the assessment of different review topics [11, 34, 40, 41]. The use of multiple assessment criteria also makes it easier for the respondents to rate the review topics as it provides clarity of what aspect of the review topic they are rating exactly. However, most RPS studies do not involve stakeholders in the selection of relevant assessment criteria [1]. We recommend to take the five stakeholder-refined assessment criteria, which we have applied (see Table 1), into consideration for other RPS studies in public health or related fields as they are based on the preferences of a broad range of public health experts.

Moreover, we found large disparities in the rating and ranking of the review topics when differentiating the results along the five assessment criteria and also along the different stakeholder groups.

The disparities between the stakeholder groups in the assessment of the review topics and in the weighting of the assessment criteria clearly show the conflicting interests of these groups and the need for including different stakeholder groups and the use of multiple assessment criteria in an RPS study [21]. The inter-criteria comparison and criteria weighting shows that distinct assessment criteria measure different dimensions of each review topic and can give insight on why a particular review topic is prioritised high or low [42]. The results corresponded to our assumption that the assessment of a particular review topic depends on the respective criterion applied (e.g., improving population health vs. insufficient research). Many RPS studies in health research do take multiple assessment criteria into rating and ranking options [11, 43], however inter-criteria differences are not presented.

Comparing the results of our RPS in Switzerland with other RPS studies that prioritised systematic review topics in public health [18, 19, 22, 43], it becomes clear that certain broader topic areas are shared top priorities, such as educational and health promotion interventions in certain settings, interventions related to access to healthcare services for vulnerable groups, and screening interventions. Furthermore, it can be observed that the top priorities are highly influenced by the geographical scope of the study. The RPS studies focusing on Nigeria [22] and West-Africa [43], prioritised several topics related to health burdens in their region, such as malaria, diarrhoea and maternal and child health, whereas our RPS and the studies of Doyle et al. [18] and Kingsland et al [19] who focused on global health issues, specifically highlighted interventions to improve mental and social health and interventions related to healthy diet and physical exercise.

### Limitations

Although the main aim of our study was to develop and implement a structured approach for prioritising review topics in public health in general, the COVID-19 pandemic has had an impact on the results of our study: The first Delphi round was conducted in 2018 before the onset of the COVID-19 pandemic and the second Delphi round was conducted in 2020 in the midst of the pandemic. Therefore, it is likely that an updated run of the first Delphi round would yield some emerging topics that were not gathered during our first Delphi round. However, we do believe that many proposed and prioritised review topics, such as “Peer-to-peer education on health risks from drugs” and “Mental health resilience training,” are still considered highly relevant. Replicating this study would help to ascertain which topics are priorities for the short-term or for the long-term.

These long-term impacts and transformational potential of research can in general be a factor when it comes to the strength of RPS studies. It is often difficult to predict what impact particular research priorities are likely to have. Unilateral or short-term prioritisation could lead to long-term challenges or potentially transformative research being neglected. It is therefore important to carefully analyse the potential long-term impact of all the topics in the RPS [14] and to simultaneously be aware that prioritising specific topics can influence the long-term direction of research within a certain field. Our structured approach can be replicated easily and therefore has the potential to also investigate if the top priorities are considered important in the long-term.

Another factor that might influence the RPS study is that our approach has only been applied in Switzerland, with unclear applicability to other countries and world regions. Also, our RPS approach is focused on prioritising topics for systematic reviews along a PICO framework only. Further investigations are needed to find out if a similar structure is applicable for prioritising topics for other types of evidence or frameworks and if this would generate very different result.

A further limitation is that we used the PICO format which may be too narrow for those public health questions that aim at the evaluation of distal changes in multi-sectoral policies with indirect impacts on health [44, 45]. Hence, a potential extension would be to ask respondents additional and more detailed questions by expanding the PICO scheme by adding further dimensions, e.g., for setting or study design. However, this would increase the burden on the respondents participating in this study while also increasing the complexity of the analysis.

Finally, we have to stress that part of the differences found between stakeholder groups might be due to the differing, and in some cases small, group sizes. In general, the validity of our sample is constrained by the specific public health organisations we identified by purposeful sampling during our stakeholder search. This, for example, led to a large standard deviation of the criteria weighting for the stakeholder group “health insurers.” Our recruitment goal was not to have exact comparable group sizes, but to establish an extensive list of stakeholders from the field of public health. An alternative to this method would have been the use of an open call for participation in the Delphi stage. However, this comes with several disadvantages, such as uncertain participation rates, potential biases, and most of all unclear levels of respondent expertise. More research is needed to fully understand the distinct priorities by stakeholder groups and if patterns exist in how different stakeholder groups prioritise review topics.

### Conclusion

Setting research priorities inherently involves value judgements and subjective decisions. These value judgements and subjective decisions may depend on the individual views, expertise and interests of the policymakers and other stakeholders [14]. The differences between stakeholder groups in the rating and ranking of the review topics and in the weighting of the assessment criteria that we found in our study clearly showed the differing interests of these groups. The contribution of a diverse range of stakeholder groups to a transparent RPS study is thus valuable in terms of content and should be encouraged through ongoing consultations with these groups in order to capitalise on the existing diversity [3].

Our applied modified Delphi technique allows for the inclusion of a large panel of respondents by presenting easy-to-conduct online surveys that reduces the workload for respondents to participate. The approach is designed to assist respondents in determining relevant systematic review topics in a PICO format without needing prior extensive knowledge on systematic reviews; thereby enabling the inclusion of all relevant stakeholders in the prioritisation process of topics for systematic reviews [46].

We believe that our RPS study can be replicated easily and likewise involve many relevant stakeholders in a prioritisation process. The online surveys and the rating results of this study are available in German, French and Italian, facilitating the adaptation and implementation of similar study designs in various settings.
