# Supplementary material for: A Structured Approach to Involve Stakeholders in Prioritising Topics for Systematic Reviews in Public Health
Source: Int J Public Health. 2024 Aug 21;69:1606642. doi: 10.3389/ijph.2024.1606642 (PMC11371559; doi:10.3389/ijph.2024.1606642)
Supplement: Supplementary file 1 [file Table1.docx]

**Supplementary File 1 – Overall score of all topics (Switzerland, 2024)**

| Review Topic | N | Mean |
| --- | --- | --- |
|  |  |  |
| Improved access to prevention services for specific target groups | 159 | 1.47 |
| Cognitive training against dementia diseases | 156 | 1.63 |
| Highlight target-group specific good practices in vulnerable groups | 163 | 1.64 |
| Better education for relatives of people with a mental illness | 166 | 1.65 |
| Mental health education for adults | 165 | 1.66 |
| More education about e-cigarettes/oral tobacco (SNUS) for children and adolescents | 156 | 1.67 |
| Peer-to-peer education on health risks from drugs | 171 | 1.69 |
| Integration with equal opportunities for chronically ill people into society | 157 | 1.71 |
| Information in schools about the correct use of mobile phones | 167 | 1.72 |
| Improving health literacy of nursing home staff in taking care of elderly people with mental disorders | 158 | 1.72 |
| Provide healthy food in schools and workplaces | 170 | 1.75 |
| Improved education about sexual health care for specific target groups | 162 | 1.75 |
| Better access to adequate care for marginalized groups | 169 | 1.76 |
| Increased early recognition structures of mental illnesses | 136 | 1.78 |
| Mental health resilience training | 161 | 1.79 |
| Interpreters to help vulnerable groups make decisions | 167 | 1.79 |
| Consultation for prevention of diabetes for risk groups | 164 | 1.79 |
| Topic “health and work” in the curriculum of medicine and human resources | 177 | 1.80 |
| More advice and support for teachers to integrate students with disabilities | 174 | 1.80 |
| Offering accessible intercultural translations for patient consultations | 176 | 1.81 |
| Improving the user-friendliness of digital health information for older people aged 80 and over | 170 | 1.81 |
| More screening of breast, colon, skin and cervical cancer for vulnerable groups | 158 | 1.81 |
| Intercultural adaptation of measures for the mental health of adolescents with a migration background | 161 | 1.81 |
| Offer of interpreting services for pregnant women with a migration background | 165 | 1.81 |
| Better access to clinical sexual health care for specific target groups | 162 | 1.81 |
| Promotion of physical activity for children with a migration background | 159 | 1.82 |
| Interpreter services for the integration of people with a migration background | 168 | 1.83 |
| Offers in the health care system to strengthen the health literacy of vulnerable groups | 164 | 1.84 |
| Implementation of practical guidelines for the care of the chronically ill | 170 | 1.84 |
| More information about harmful drug combinations for prescribing doctors | 165 | 1.86 |
| Empowerment for self-management in multimorbidity | 172 | 1.87 |
| Integration of caring relatives into the health care system | 161 | 1.87 |
| Restricting children's access to unhealthy foods | 180 | 1.87 |
| More low-threshold counseling services on the mental health of children and young people | 158 | 1.87 |
| Self-management for chronically ill people | 165 | 1.87 |
| Home care for relatives of the mentally ill | 163 | 1.88 |
| Obesity centers for children and young people with interdisciplinary cooperation between doctors | 155 | 1.88 |
| Expanding mass screening PSA (prostate-specific-antigen) test for prostate cancer | 151 | 1.90 |
| Support of group practices with interprofessional cooperation | 164 | 1.90 |
| Creating access for people in precarious work situations and the unemployed | 165 | 1.90 |
| Designing the transition from home to a retirement home | 160 | 1.91 |
| Outreach visits to vulnerable groups | 155 | 1.91 |
| Strengthening the health skills of flexible workers in the face of stress and uncertainty | 160 | 1.93 |
| Development of the know-how of carers in schools for the integration of chronically ill children | 169 | 1.93 |
| More information/education on healthy exercise and nutrition for immigrants | 159 | 1.93 |
| Expansion of infrastructure for bicycles | 164 | 1.95 |
| Development of the health competence of relatives with regard to dealing with old people with mental disorders | 159 | 1.95 |
| Education campaigns for young people regarding the health risk of smoking | 168 | 1.95 |
| Walk-in practices for preliminary clarifications | 161 | 1.96 |
| Self-help groups to strengthen the health literacy of patients | 158 | 1.96 |
| Promoting the social participation of older people | 160 | 1.96 |
| Sustaining the weight loss of obese people | 163 | 1.96 |
| Subsidies for healthy food | 171 | 1.98 |
| More safety advice on accident prevention for older people | 161 | 1.98 |
| Raising awareness among health professionals about the psychological and social dimensions of health | 164 | 1.99 |
| Incentive to maintain physical activity for workers | 156 | 1.99 |
| More early screening to detect developmental abnormalities | 168 | 1.99 |
| Educating doctors and pharmacists about obesity | 162 | 1.99 |
| Funding of preventive measures through basic insurance | 157 | 1.99 |
| Better inpatient care for mental illnesses in the perinatal period | 157 | 2.01 |
| Use of telecare in outpatient care | 159 | 2.01 |
| Expansion of the school medical service for early detection of children and adolescents | 156 | 2.01 |
| More health information and offers to reach vulnerable groups | 171 | 2.01 |
| Support groups for older people regarding exercise | 166 | 2.02 |
| Financial support for poor families | 168 | 2.02 |
| Establishment of good and effective cooperation in the supply chain | 192 | 2.02 |
| Funding for peer services to care for sick people | 174 | 2.02 |
| Better access to perinatal care for refugee and migrant women | 179 | 2.02 |
| Expansion of home infrastructure to prevent falls for the elderly | 168 | 2.03 |
| Substitution of medical services in health centers | 154 | 2.04 |
| Self-help groups for people with a migration background | 165 | 2.04 |
| Training / more support for patients with serious health problems | 157 | 2.04 |
| Financial support/ donations/ minimum integration income for disadvantaged people | 168 | 2.05 |
| "Blended Treatment" to strengthen the social skills of young people | 161 | 2.05 |
| Measures to improve the nutrition of children with a migration background | 168 | 2.05 |
| Social and cultural measures focused on social participation of older people | 157 | 2.05 |
| Supporting mothers to promote resilience in children | 163 | 2.06 |
| Better accessibility to digital health information for older people aged 80+ | 173 | 2.07 |
| Strengthening of skills for school integration of disadvantaged children and young people | 156 | 2.07 |
| Food banks with healthy food for disadvantaged groups | 165 | 2.08 |
| Inclusion of measurements carried out by the client in outpatient care | 179 | 2.08 |
| Providing positive psychology in schools for child and youth resilience | 174 | 2.08 |
| Raising awareness and breaking the taboo on domestic violence in families | 162 | 2.08 |
| More understandable communication of scientific findings to adolescents and young adults | 161 | 2.09 |
| Clearer information on strengthening the health literacy of vulnerable groups | 173 | 2.09 |
| Training of medical professionals and journalists in the assessment of medical data | 160 | 2.09 |
| Warning labels on alcohol products (like cigarettes) | 160 | 2.09 |
| Acceptance of the changed food supply | 161 | 2.10 |
| Individualized care of vulnerable patients in health care | 166 | 2.10 |
| Better support for young adults with autism to avoid psychiatric and behavioral disorders | 166 | 2.10 |
| Design of a living environment conducive to physical activity | 156 | 2.11 |
| Peer-to-peer violence prevention programs among high school students | 169 | 2.11 |
| Implementation of a communication network for the care of the chronically ill | 158 | 2.11 |
| Nutritional coaching for parents of kindergarten children | 168 | 2.11 |
| Establishment of advanced practice roles in healthcare professions | 170 | 2.11 |
| Strengthening the health literacy of young women with regard to nutrition | 174 | 2.12 |
| Interventions to reduce health inequalities, e.g. B. Guaranteed Minimum Income | 177 | 2.12 |
| Systematic recording of somatic and mental illnesses of asylum-seeking women of reproductive age | 161 | 2.12 |
| Better access to good quality clinics for vulnerable groups in poor countries | 163 | 2.12 |
| More screening of patients with angina pectoris with suspected coronary artery disease | 177 | 2.12 |
| Access to relevant information on the quality and quantity of food | 166 | 2.13 |
| Establishing training courses for doctors to talk to patients | 187 | 2.13 |
| Parental support for early support for disadvantaged children and young people | 156 | 2.13 |
| Higher funding for outreach-help for the mentally ill in acute situations | 171 | 2.13 |
| Help for patients to actively participate in decisions | 164 | 2.14 |
| Low Method (Positive Reinforcement) in the field of nutrition | 169 | 2.14 |
| Student-for-student workshops on dealing with digital media | 162 | 2.15 |
| Fairness courses to prevent violence among students | 162 | 2.15 |
| Improving monitoring and prescribing practice in everyday life for the chronically ill | 174 | 2.16 |
| More campaigns/training programs for athletes against sports injuries | 171 | 2.16 |
| Strengthening the health literacy of young women with regard to alcohol and nicotine consumption | 158 | 2.16 |
| Strengthening of resilience skills against unexpected psychological stress in adolescents and young adults | 158 | 2.16 |
| Offering sexual and reproductive health programs to increase the health literacy of asylum-seeking women | 165 | 2.16 |
| Linking inpatient and outpatient facilities for the aftercare of premature babies | 161 | 2.17 |
| Identification of vulnerable target groups | 157 | 2.17 |
| More recommendation to avoid sudden infant death syndrome | 161 | 2.17 |
| Reducing stress against social isolation among migrants | 164 | 2.17 |
| Dietary reduction of sugar and/or salt | 158 | 2.17 |
| Raising awareness in schools about the use of different means of transport | 168 | 2.18 |
| Introduction of minimum case numbers in Swiss hospitals | 162 | 2.18 |
| More screening for common chronic diseases | 177 | 2.19 |
| Expansion of mass screening of mammography for breast cancer | 173 | 2.20 |
| Training/ more support for the relatives of patients with serious health problems | 165 | 2.20 |
| Employment and contact-offers to relieve the mentally ill and their relatives | 165 | 2.20 |
| Strengthening the social skills of children and young people with regard to conflict management | 174 | 2.20 |
| Better access to health care providers for transgender and non-binary people | 170 | 2.21 |
| Vaccination campaigns in schools | 163 | 2.21 |
| Decelerating the medical care of pregnant women by specialists | 163 | 2.21 |
| Training programs for obstetricians/midwives | 160 | 2.23 |
| Education and training program for pregnant/parturient women | 155 | 2.23 |
| Use of the electronic maternity record with continuous information | 154 | 2.23 |
| More substitution programs for older people | 173 | 2.24 |
| Expansion of support services for children and families affected by poverty | 163 | 2.24 |
| Incorporating health education into the school curriculum in low-income countries | 165 | 2.24 |
| Introduction of an electronic patient dossier in paediatrics | 166 | 2.25 |
| Inclusion of vulnerable groups in measures to prevent viral diseases | 161 | 2.25 |
| Training for children and young people in dealing with life crises | 161 | 2.25 |
| More time for exercise in schools and at work | 162 | 2.25 |
| More Blended Treatment of Mental Disorders | 170 | 2.25 |
| More prevalence data of mental illnesses in the perinatal period | 168 | 2.26 |
| Care navigation in the healthcare system for the chronically ill | 174 | 2.26 |
| Use of an online registration system in outpatient care | 170 | 2.26 |
| Better education of children and young people on self-reflection regarding health | 161 | 2.26 |
| Providing access to food for older people at home | 172 | 2.26 |
| Strengthening the social skills of children and young people against school failure | 162 | 2.26 |
| Medical assessment of fitness to drive seniors to prevent accidents | 154 | 2.27 |
| Establishment of competence centers for patients with rare diseases | 162 | 2.27 |
| More efficient use of staff in non-physician health professions | 173 | 2.27 |
| Convincing communication from medical staff to parents about vaccinations | 176 | 2.27 |
| Better access to physical activity programs for patients at risk of falls | 163 | 2.28 |
| Stress prevention for family members of the mentally ill | 154 | 2.28 |
| Follow-up of the medical care of patients | 152 | 2.28 |
| Use of new media application techniques, newsletters on healthy behaviors | 170 | 2.28 |
| Self-help groups for older people regarding nutrition | 177 | 2.28 |
| More low-threshold counseling services for people with mental health problems | 164 | 2.29 |
| More outpatient and semi-inpatient offers for people with mental disorders | 162 | 2.29 |
| Promoting body knowledge in women during pregnancy, childbirth and postpartum | 161 | 2.30 |
| Establishment of a seal of quality/a clear mark for reliable health communication | 161 | 2.30 |
| Revaluation of the care time of a family member in the pension insurance | 161 | 2.31 |
| Early occupational therapy intervention in people after stroke | 179 | 2.32 |
| Promotion of physical activity in everyday life for adolescents and young adults | 164 | 2.32 |
| Health education on brain physiology and overeating | 163 | 2.33 |
| Job security for family members of the mentally ill | 160 | 2.33 |
| Monitoring of addictions in adolescents and adults | 160 | 2.33 |
| Strengthening of people's health competence for risk situations in nature | 169 | 2.34 |
| Strengthening of the physical requirements for physically demanding jobs | 163 | 2.35 |
| More monitoring of venereal diseases of specific target groups | 171 | 2.35 |
| Better room design in schools to integrate students with disabilities | 182 | 2.35 |
| Establishment of an independent monitoring system in outpatient care | 182 | 2.35 |
| Better help in case of illness for single people | 166 | 2.36 |
| Adjusting the training of psychiatrists to understand neurodevelopmental disorders | 170 | 2.36 |
| Changing portion sizes to promote a balanced diet | 163 | 2.36 |
| Training medical staff to communicate verbally with patients in poor countries | 172 | 2.36 |
| More participation in screening | 177 | 2.37 |
| Financial incentive for carers / families of the mentally ill | 164 | 2.37 |
| Ban on tobacco advertising and sponsorship | 164 | 2.37 |
| Mandatory labeling for fat and sugar in food | 160 | 2.38 |
| Free exercise offers for migrants | 161 | 2.39 |
| Offer of a neutral contact point for relatives of mentally ill people | 187 | 2.39 |
| Establishment of conflict pilots to prevent violence among schoolchildren | 158 | 2.40 |
| Low-threshold employment offers in intergenerational contact for children, young people, senior citizens and social welfare recipients | 177 | 2.40 |
| Implementation of a case manager as a contact person for complex cases of illness | 173 | 2.40 |
| Person-centred care without staff turnover for the mentally ill | 162 | 2.40 |
| Delivery of sterile consumables to inmates | 169 | 2.40 |
| Decoupling of indication and treatment by healthcare providers | 173 | 2.40 |
| Establishment of information campaigns regarding midwife-led births for women of reproductive age | 171 | 2.41 |
| Promotion of physical activity for seniors to prevent falls | 176 | 2.41 |
| Offer of occupational therapy to improve the social participation of older people | 170 | 2.41 |
| Parental orders for adolescents to be withdrawn from addiction without their consent | 175 | 2.42 |
| Avoiding unnecessary cesarean sections | 157 | 2.42 |
| Physical activity monitoring | 170 | 2.42 |
| Active lessons for school children against back pain | 180 | 2.43 |
| Establishment of individual computer-protected coaching systems to promote physical activity | 174 | 2.43 |
| Better monitoring of aftercare for vulnerable groups | 162 | 2.44 |
| National Weight Loss Registry for Adult Volunteers | 166 | 2.45 |
| Promotion of physiology during pregnancy, childbirth, postpartum | 168 | 2.46 |
| Education of the population to destigmatize dementia | 162 | 2.48 |
| Accessibility of migrants with an addiction problem | 170 | 2.49 |
| Systematic assessment of the health needs of asylum-seeking women of reproductive age | 163 | 2.50 |
| Supported employment for the reintegration of people with mental health problems | 173 | 2.50 |
| Midwife-led preventive care and control of pregnant women | 166 | 2.50 |
| Improving air quality in big cities | 163 | 2.50 |
| Use of generic/over-the-counter medications | 175 | 2.50 |
| Improving job satisfaction in healthcare | 170 | 2.51 |
| Audits of obstetric interventions | 158 | 2.52 |
| Introduction of screening for psychosocial risk factors in women who have recently given birth | 176 | 2.52 |
| Screening of prevention standards in prison medicine | 165 | 2.52 |
| Low-threshold exercise courses for kindergarten children | 155 | 2.52 |
| Quality assurance programs for obstetricians/midwives | 164 | 2.54 |
| Hearing and sight tests for refugee children who enter through the Zurich asylum organization | 173 | 2.54 |
| Empowering communities to strengthen the health literacy of vulnerable groups | 178 | 2.54 |
| Legalization of cannabis for young people up to the age of 30 | 165 | 2.55 |
| Fitness center with professional guidance especially for adolescents | 158 | 2.55 |
| Shift from car driving to other modes of transport among older people | 172 | 2.55 |
| More specially trained family care professionals for the mentally ill | 168 | 2.55 |
| Subsidies for the provision of inclusive workplaces for burdened and stress-sensitive people | 169 | 2.56 |
| Increase in tobacco tax | 163 | 2.56 |
| Schwingen (Swiss wrestling) to prevent violence among schoolchildren | 164 | 2.57 |
| Create health-promoting conditions for families in which both parents work | 160 | 2.57 |
| More monitoring of environmental pollution | 164 | 2.57 |
| Politically emphasize noise abatement measures | 180 | 2.59 |
| More information and support for changing the behavior of pregnant women | 158 | 2.59 |
| Stress management strategies for children and adolescents | 160 | 2.59 |
| Development of the skills of older people in dealing with support and care authorities | 176 | 2.59 |
| Providing housing for the homeless | 164 | 2.60 |
| Sugar tax | 163 | 2.60 |
| Offer regional outreach psychiatry and home care | 178 | 2.60 |
| Establishment of a personalized treatment with accelerated approved drugs | 155 | 2.62 |
| Ergonomic school furniture against back pain in school children | 178 | 2.62 |
| Health education regarding psychomodulators and long-term psychological effects | 163 | 2.65 |
| Vegan diet for teenagers and adults | 164 | 2.66 |
| Possibility of childcare outside the home to support women/families | 160 | 2.67 |
| Exercise offers for the chronically ill | 174 | 2.68 |
| Avoiding unnecessary labor inductions | 159 | 2.69 |
| Establishment of self-help-friendly health institutions for better networking with NGOs | 154 | 2.70 |
| Autonomous driving for accident prevention | 167 | 2.71 |
| Support for partners incorporated in national addiction-strategies | 160 | 2.71 |
| More continuing education programs in health care for gynecologists | 152 | 2.72 |
| Craniosacral therapy for regulatory disorders in newborns | 160 | 2.74 |
| "Health" as a subject in schools for folic acid prophylaxis | 161 | 2.76 |
| Avoiding unnecessary oxytocin to stimulate labor | 166 | 2.79 |
| Promotion of obstetricians to avoid unnecessary caesarean sections | 173 | 2.93 |
| Nutritional supplements during pregnancy | 160 | 2.93 |
| Promotion of obstetricians to avoid unnecessary oxytocin stimulation | 153 | 2.97 |
| Better release binding for skis | 154 | 3.08 |
|  |  |  |
|  |  |  |
