# Supplementary material for: A Structured Approach to Involve Stakeholders in Prioritising Topics for Systematic Reviews in Public Health
Source: Int J Public Health. 2024 Aug 21;69:1606642. doi: 10.3389/ijph.2024.1606642 (PMC11371559; doi:10.3389/ijph.2024.1606642)
Supplement: Supplementary file 2 [file Table2.docx]

**Supplementary File 2 – Best rated topics per criterion (Switzerland, 2024)**

3.1. Best rated review topics. Criterion: “Improving the health of population”

| **Rank** | **Topic** | **N** | **Mean** |
| --- | --- | --- | --- |
| 1 | More education about e-cigarettes/oral tobacco (SNUS) for children and adolescents | 34 | 1.34 |
| 2 | Self-management for chronically ill people | 35 | 1.40 |
| 3 | Improved access to prevention services for specific target groups | 32 | 1.41 |
| 4 | Topic “health and work” in the curriculum of medicine and human resources | 32 | 1.43 |
| 5 | Support for groups practices with interprofessional collaboration | 34 | 1.44 |
| 6 | Improved education about sexual health care for specific target groups | 33 | 1.45 |
| 7 | Consultation for prevention of diabetes for risk groups | 37 | 1.46 |
| 8 | Cognitive training against dementia diseases | 33 | 1.48 |
| 9 | Developing the health skills of nursing home staff in dealing with elderly people with mental disorders | 33 | 1.52 |
| 10 | Mental health resilience training | 39 | 1.54 |
| 11 | Improved education for family members of mentally ill people | 34 | 1.55 |
| 12 | Sustaining the weight loss of obese people | 34 | 1.56 |
| 13 | Offers in the health care system to strengthen the health literacy of vulnerable groups | 33 | 1.56 |
| 14 | Mental health education for adults | 32 | 1.59 |
| 15 | More early detection of mental illnesses | 32 | 1.59 |

3.2. Best rated review topics. Criterion: “Health equity”

| **Rank** | **Topic** | **N** | **Mean** |
| --- | --- | --- | --- |
| 1 | Highlight target group specific good practices in vulnerable groups | 33 | 1.19 |
| 2 | Improved access to prevention services for specific target groups | 33 | 1.21 |
| 3 | More education about e-cigarettes/oral tobacco (SNUS) for children and adolescents | 30 | 1.30 |
| 4 | Better inpatient care for mental illnesses in the perinatal period | 30 | 1.30 |
| 5 | Funding of preventive measures through basic insurance | 33 | 1.34 |
| 6 | Offers in the health care system to strengthen the health literacy of vulnerable groups | 34 | 1.35 |
| 7 | Better education for family members of mentally ill people | 45 | 1.38 |
| 8 | Interpreter services for the integration of people with a migration background | 39 | 1.38 |
| 9 | Improved education about sexual health care for specific target groups | 36 | 1.39 |
| 10 | Intercultural adaptation of measures for the mental health of adolescents with a migration background | 33 | 1.39 |
| 11 | Interpreters to help vulnerable groups make decisions | 34 | 1.41 |
| 12 | Strengthening of skills for school integration of disadvantaged children and young people | 33 | 1.41 |
| 13 | Promotion of physical activity for children with a migration background | 36 | 1.42 |
| 14 | Consultation for prevention of diabetes for risk groups | 35 | 1.44 |
| 15 | Better access to appropriative care for marginalized groups | 38 | 1.46 |

3.3. Best rated review topics. Criterion: ““Insufficient research to date (e.g., are not available, are of insufficient quality, are not up to date or will become more important in the future)”

| **Rank** | **Topic** | **N** | **Mean** |
| --- | --- | --- | --- |
| 1 | Cognitive training against dementia diseases | 34 | 1.32 |
| 2 | Supporting mothers to promote resilience in children | 35 | 1.43 |
| 3 | Mental health resilience training | 32 | 1.46 |
| 4 | Mental health education for adults | 32 | 1.47 |
| 5 | Improved access to prevention services for specific target groups | 34 | 1.48 |
| 6 | Integration of caring relatives into the health care system | 33 | 1.54 |
| 7 | Training of medical professionals and journalists in the assessment of medical data | 37 | 1.55 |
| 8 | Use of telecare in outpatient care | 33 | 1.56 |
| 9 | Better education for family members of mentally ill people | 33 | 1.57 |
| 10 | Acceptance of the changed food supply | 39 | 1.58 |
| 11 | Incentive to maintain physical activity for workers | 34 | 1.60 |
| 12 | Individualized care of vulnerable patients in health care | 34 | 1.64 |
| 13 | More participation in screening | 33 | 1.64 |
| 14 | Strengthening the health skills of flexible workers in the face of stress and uncertainty | 32 | 1.65 |
| 15 | Developing the knowledge of carers in schools for the integration of chronically ill children | 32 | 1.66 |

3.4. Best rated review topics. Criterion: “Effect on public health if successful”

| **Rank** | **Topic** | **N** | **Mean** |
| --- | --- | --- | --- |
| 1 | Better access to good quality hospitals for vulnerable groups in poor countries | 35 | 1.32 |
| 2 | Integration with equal opportunities for chronically ill people into society | 39 | 1.36 |
| 3 | Creating access for people in precarious work situations and unemployed people | 30 | 1.36 |
| 4 | More safety advice on accident prevention for elderly people | 31 | 1.42 |
| 5 | More outpatient and semi-inpatient offers for people with mental disorders | 31 | 1.43 |
| 6 | More health information and offers to reach vulnerable groups | 34 | 1.44 |
| 7 | Quality assurance programs for obstetricians/midwives | 45 | 1.46 |
| 8 | More screening of breast, colon, skin and cervical cancer for vulnerable groups | 39 | 1.47 |
| 9 | More social and cultural interventions focused on social participation of elderly people | 36 | 1.47 |
| 10 | More substitution programs for elderly people | 33 | 1.47 |
| 11 | More early detection of mental illnesses | 34 | 1.50 |
| 12 | Emphasize target group specific good practices in vulnerable groups | 31 | 1.50 |
| 13 | Strengthening the health literacy of young women regarding alcohol and nicotine consumption | 36 | 1.52 |
| 14 | More information/ education on health exercises and healthy diets for immigrants | 35 | 1.52 |
| 15 | Mental health education for adults | 38 | 1.53 |
|  | Better inpatient care for mental illnesses in the perinatal period | 30 | 1.53 |

3.5. Best rated review topics. Criterion: “Potential for innovative action”

| **Rank** | **Topic** | **N** | **Mean** |
| --- | --- | --- | --- |
| 1 | Support for group practices with interprofessional collaboration | 31 | 1.35 |
| 2 | Better access to prevention services for specific target groups | 35 | 1.37 |
| 3 | Peer-to-peer education on health risks from drugs | 30 | 1.44 |
| 4 | Expanding mass screening PSA (prostate-specific-antigen) test for prostate cancer | 32 | 1.44 |
| 5 | More information about harmful drug combinations for prescribing doctors | 39 | 1.50 |
| 6 | Educating doctors and pharmacists about obesity | 33 | 1.50 |
| 7 | Developing the health skills of nursing home staff in dealing with elderly people with mental disorders | 39 | 1.55 |
| 8 | Audits of obstetric interventions | 33 | 1.55 |
| 9 | Interventions to reduce health inequalities, e.g., guaranteed minimum wage | 33 | 1.55 |
| 10 | Mental health education for adults | 39 | 1.59 |
| 11 | Better access to appropriate care for marginalized groups | 30 | 1.59 |
| 12 | More campaigns/ training programs for athletes to prevent sports injuries | 34 | 1.60 |
| 13 | More early detection of mental illness | 30 | 1.61 |
| 14 | Strengthening of resilience skills against unexpected psychological stress in adolescents and young adults | 37 | 1.61 |
| 15 | Cognitive training against dementia diseases | 32 | 1.63 |
